# Serum uric acid levels and prognosis of patients with non-alcoholic fatty liver disease

**DOI:** 10.1038/s41598-024-55845-5

**Published:** 2024-03-11

**Authors:** Xinyi Yang, Yan Lin, Jiaofeng Huang, Yujing Chi, Yinlian Wu, Su Lin

**Affiliations:** 1 Department of Hepatology, Hepatology Research Institute, the First Affiliated Hospital, Fujian Medical University, No. 20, Chazhong Road, Taijiang District, Fuzhou, 350005 Fujian China; 2 Fujian Clinical Research Center for Liver and Intestinal Diseases, Fuzhou, 350002 Fujian China; 3 Nursing Treatment Center, The First Affiliated Hospital, Fujian Medical University, Fuzhou, China

**Keywords:** NAFLD, UA, Mortality, NHANES, Biomarkers, Diseases, Medical research

## Abstract

Uric acid (UA) is associated with non-alcoholic fatty liver disease (NAFLD). However, it is unclear whether UA plays a predictive role in NAFLD prognosis. This study aimed to explore the relationship between UA levels and mortality in NAFLD patients without severe renal disease. Data were obtained from the Third National Health and Nutrition Examination Survey (NHANES). Time-dependent Cox regression was used to estimate the hazard ratio (HR) and 95% confidence interval (CI) for mortality. Overall, 2493 individuals with NAFLD and estimated glomerular filtration rate (eGFR) > 60 mL/min/1.73 m^2^ were included in this study. The median follow-up period was 26.58 years. Patients were divided into high and low-UA groups according to UA levels. Time-independent Cox regression showed that UA level was not an independent risk factor for mortality in NAFLD patients without decreased eGFR (*P* > 0.05). After matching for age and sex using the propensity score matching method, UA remained not independently associated with death in NAFLD patients (*P* > 0.05). Similar results were found for cardiovascular-related and cancer-related deaths. Although UA is closely related to NAFLD, UA levels are not independently associated with the long-term survival of patients with NAFLD without decreased eGFR.

## Introduction

NAFLD is characterised by excessive hepatic fat accumulation, associated with insulin resistance (IR), and defined by the presence of steatosis in > 5% of hepatocytes according to histological analysis or by a proton density fat fraction (PDFF, providing a rough estimation of the volume fraction of fatty material in the liver) > 5.6% assessed by proton magnetic resonance spectroscopy or quantitative fat/water selective magnetic resonance imaging (MRI)^1^. It is the most common liver disease worldwide, with a global prevalence of 25%^2^. Metabolic factors, such as obesity, type 2 diabetes, hyperlipidaemia, and hypertension, Sugar-sweetened beverages intake and a history of obesity in their early adulthood are closely associated with NAFLD and may accelerate its progression^3–5^.

Uric acid (UA) is the final product of purine metabolism. Hyperuricaemia is usually caused by a reduction in the renal excretion and/or overproduction of UA. Therefore, hyperuricaemia is often observed in patients with renal or metabolic diseases^6^. Previous studies have shown a significant association between hyperuricaemia and NAFLD^7–9^. For example, Li et al. demonstrated a higher prevalence of NAFLD in patients with hyperuricaemia than in those without^7^. Petta et al. showed that hyperuricaemia is independently associated with the severity of liver damage in NAFLD^10^. However, most previous studies have been cross-sectional, and the relationship between UA levels and NAFLD outcomes has not been fully explored. This study aimed to evaluate the role of UA levels in predicting the outcome of NAFLD in a large population-based survey cohort.

## Method

### Ethics

The study data were obtained from the Third National Health and Nutrition Examination Survey, a major nationwide survey conducted in the United States between 1988 and 1994. All study subjects were followed up until December 2015 to determine their survival status or cause of death. The study dataset was uploaded online for researchers worldwide. All datasets used in this study were obtained from the NHANES website (https://www.cdc.gov/nchs/nhanes/index.htm). This study was approved by the Institutional Review Board of the First Affiliated Hospital of Fujian Medical University (IEC-FOM-013-2.0).

The inclusion criteria for this study were adults with ultrasonography-confirmed fatty liver disease. The assess of hepatic steatosis was obtained by re-reviewing the archived gall bladder ultrasound video images derived from NHANES III between 1988 and 1994.

Patient exclusion criteria were as follows: 1. lost to follow-up; 2. missing data; 3. positive for HBsAg or anti-HCV antibody; 4. excessive alcohol consumption; and 5. decreased glomerular filtration rate (GFR), i.e., with estimated GFR (eGFR) < 60 mL/min/1.73 m^2^. eGFR was calculated according to the 2009 CKD-EPI equation as follows: GFR = 141 min(Scr/κ, 1)^α^ × max(Scr/κ, 1)^–1.209^ × 0.993^Age^ × 1.018 (if female) × 1.159 (if black), where Scr is serum creatinine, κ is 0.7 for females and 0.9 for males, α = –0.329 for females and –0.411 for males^11^.

### Statistical analysis

Continuous variables are expressed as mean ± standard deviation and compared using the Student’s t-test. Categorical variables are displayed as percentages and compared using the chi-square test. X-tile (Version3.6.1, http://tissuearray.org/) was used to determine the best UA cut-off value to discriminate between survival and death. A Kaplan–Meier survival curve was plotted to illustrate mortality between cohorts using R software (https://www.r-project.org/). Time-dependent Cox models were used to assess hazard ratios (HRs) and 95% confidence intervals (CI). Propensity score matching (PSM) was applied to match age and sex, to balance the baseline characteristics between the two study groups. All tests were two-tailed, and results with a *P* value less than 0.05 were considered statistically significant. The statistical analysis was conducted using SPSS 22.0 (SPSS Inc., Chicago, IL, USA).

### Ethical approval

This study was supported by Fund for nurse innovation research (No. 2022FY-HZ-20) and Natural Science Fundation of Fujian Province (No. 2023J01580). All methods performed in this study were carried out in accordance with the Declaration of Helsinki and approved by the the National Center for Health Statistics Ethics Review Board. Informed consent was obtained from all participants involved in the study.

## Results

### Baseline participant characteristics

**Figure 1:**
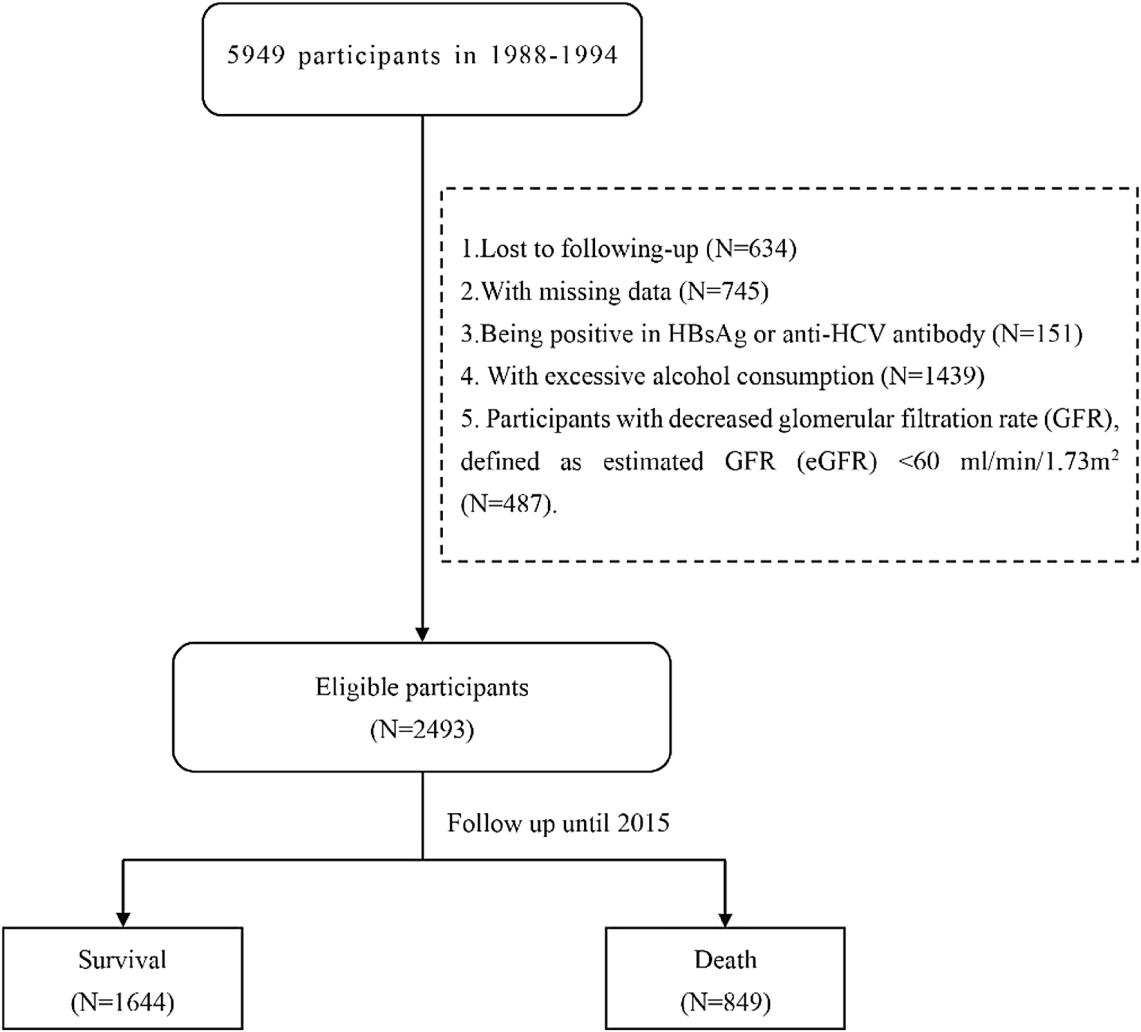
Case selection flowchart.

A total of 5949 adults with hepatic steatosis were screened. After excluding patients lost to follow-up (N = 634), with missing data (N = 745), positive for HBsAg or anti-HCV antibody (N = 151), excessive alcohol consumption (N = 1439), and decreased eGFR (N = 487), 2493 individuals with NAFLD remained eligible for final analysis (Fig. 1). Of these, 1410 (56.56%) were male, and the mean age was 44.21 ± 15.03 years. Diabetes and hypertension were diagnosed in 150 (17.69%) and hypertension in 1270 (50.94%) patients, respectively. The mean body mass index (BMI) level was 28.93 ± 6.83 kg/m^2^. The mean UA level was 311.94 ± 85.56 μmol/L, and 44.89% of cases had levels greater than 320 μmol/L.

### Survival versus death groups

The median follow-up period was 26.58 (22.46, 28.42) years. During this period, 849 (34.06%) deaths were recorded. The baseline characteristics of the survival and death groups are presented in Table 1. Compared to those in the survival group, patients in the death group were significantly older with higher BMI. The metabolic profiles, including fasting plasma glucose, glycated haemoglobin, total cholesterol, and triglycerides, were higher in the death versus the survival group. There were more cases of hypertension in the death group. Patients who died were more likely to have more elevated renal function parameters (creatinine and urea nitrogen). The mean UA level was 325.63 ± 86.60 in the death group, which was significantly higher than that of the survival group (304.86 ± 84.17, *P* < 0.001). More individuals had UA levels greater than 320 μmol/L in the death group versus the survival group (52.53% vs. 40.94%, *P* < 0.001).

**Table 1:** Comparison of survival and death groups.

Variables	Total	Survival	Death	*p*
N	2493	1644	849	
Follow years	24.04 ± 6.98	27.84 ± 1.68	16.67 ± 7.43	< 0.001
Age (years)	44.21 ± 15.03	37.97 ± 12.03	56.29 ± 12.72	< 0.001
Male (%)	1410 (56.56)	1017 (61.86)	393 (46.29)	< 0.001
BMI (Kg/m^2^)	28.93 ± 6.83	28.49 ± 6.80	29.78 ± 6.81	< 0.001
Waist-to-hip ratio	0.93 ± 0.09	0.91 ± 0.09	0.98 ± 0.08	< 0.001
Race, n (%)				< 0.001
Non-Hispanic White	801 (32.13)	476 (28.95)	325 (38.28)	
Non-Hispanic Black	655 (26.27)	432 (26.28)	223 (26.27)	
Mexican–American	926 (37.14)	649 (39.48)	277 (32.63)	
Other	111 (4.45)	87 (5.29)	24 (2.83)	
Fasting plasma glucose (mmol/L)	5.86 ± 2.61	5.45 ± 1.98	6.64 ± 3.40	< 0.001
Glycated haemoglobin (%)	5.74 ± 1.40	5.49 ± 1.15	6.23 ± 1.68	< 0.001
Total Cholesterol (mmol/L)	5.31 ± 1.13	5.15 ± 1.09	5.61 ± 1.16	< 0.001
Triglycerides (mmol/L)	1.83 ± 1.34	1.71 ± 1.31	2.06 ± 1.36	< 0.001
HDL (mmol/L)	1.24 ± 0.37	1.25 ± 0.36	1.21 ± 0.38	0.032
Hypertension, n (%)	1270 (50.94)	695 (42.27)	575 (67.73)	< 0.001
Diabetes, n (%)	150 (17.69)	0.00	150 (17.69)	1.000
AST (U/L)	22.39 ± 12.19	22.4 ± 12.73	22.38 ± 11.08	0.974
ALT (U/L)	20.24 ± 15.49	21.06 ± 16.72	18.65 ± 12.60	< 0.001
eGFR (mL/min/1.73 m^2^)	83.29 ± 15.93	86.55 ± 15.95	76.98 ± 13.87	< 0.001
Creatinine (μmol/L)	88.50 ± 15.86	87.81 ± 15.99	89.86 ± 15.52	0.002
Urea nitrogen (mmol/L)	4.69 ± 1.49	4.55 ± 1.41	4.96 ± 1.58	< 0.001
UA (μmol/L)	311.94 ± 85.56	304.86 ± 84.17	325.63 ± 86.60	< 0.001
UA > 320 μmol/L, n (%)	1119 (44.89)	673 (40.94)	446 (52.53)	< 0.001

*NAFLD* non-alcoholic fatty liver disease, *UA* uric acid, *NHANES III* Third National Health and Nutrition Examination Survey, *eGFR* estimated glomerular filtration rate, *BMI* body mass index, *WHR* Waist-to-hip ratio, *FPG* fasting plasma glucose, *HBA1C* glycated haemoglobin, *TCHO* total cholesterol, *TG* triglycerides, *HDL* high-density lipoprotein cholesterol, *CR* creatinine, *BUN* blood urea nitrogen, *AST* aspartate transaminase, *ALT* alanine aminotransferase.

### High versus low-UA groups

**Figure 2:**
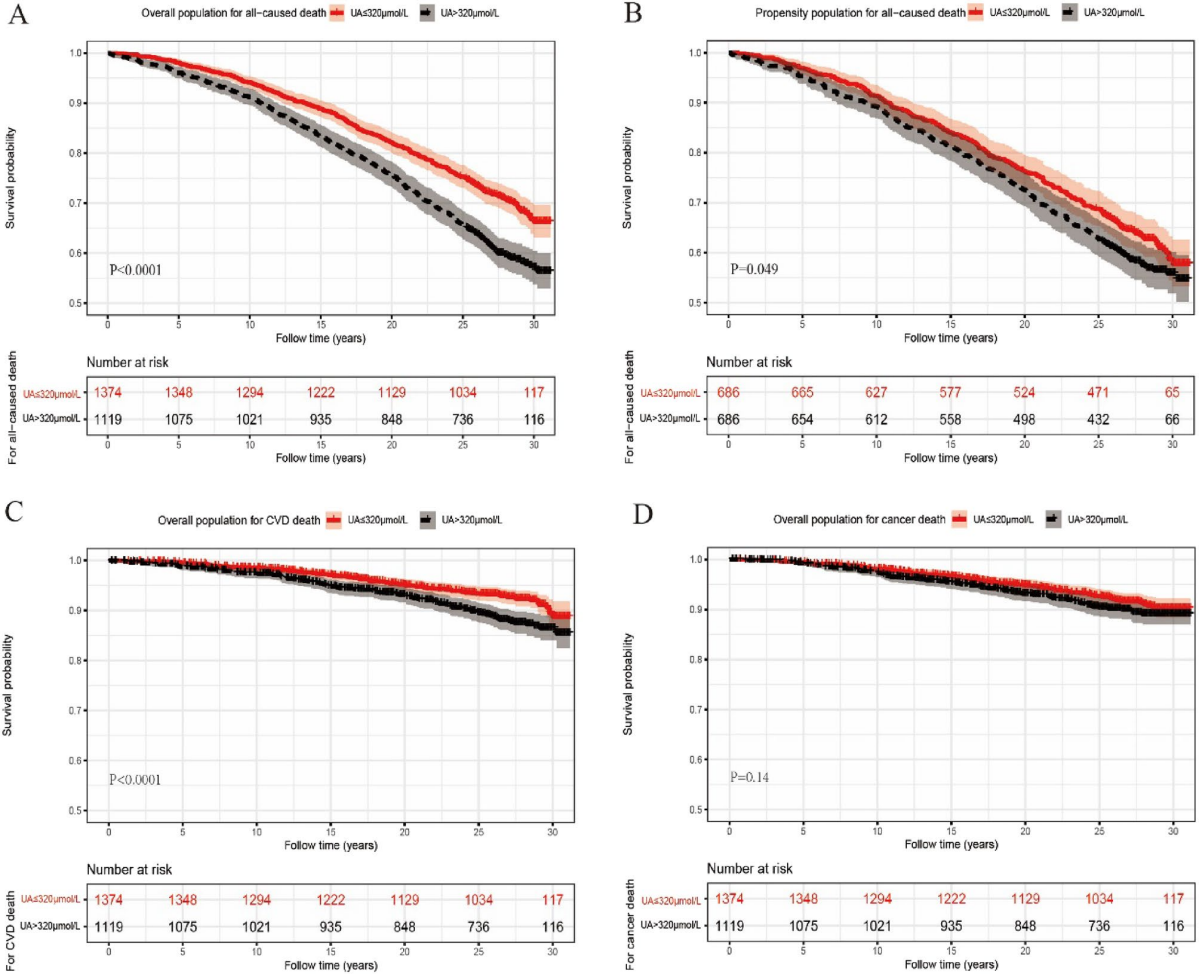
(**A**) Kaplan–Meier curve for overall death before PSM. (**B**) Kaplan–Meier curve for overall death after PSM. (**C**) Kaplan–Meier curve for cardiovascular-related death. (**D**) Kaplan–Meier curve for cancer-related death.

We used the X-tile software to determine the best UA cut-off value for assessing outcome. The results showed that at 320 μmol/L, serum UA level predicted the long-term survival of patients with NAFLD. A comparison of the high (> 320 μmol/L) and low (≤ 320 μmol/L) UA groups is presented in Table 2. The high-UA group was older and more likely to have increased BMI. Liver and renal injuries, evident by aspartate transaminase, alanine aminotransferase, creatinine, and eGFR, were more severe in the high versus low-UA group. Metabolic indices, such as total cholesterol and triglyceride levels, were significantly elevated in the high-UA group, whereas the low-UA group had higher high-density lipoprotein cholesterol levels. The death rate was 39.86% in the high-UA group, compared to 29.33% in the low-UA group. The survival probability between the two UA groups is illustrated as a Kaplan–Meier curve in Fig. 2A, indicating the association between baseline high UA levels and risk of death (*P* < 0.001). In the mild- and moderate group, patients with UA > 320 μmol/L had a higher risk of all-cause death (Supplementary Fig. 1A,B), while no difference was found in severe fatty liver group (Supplementary Fig. 1C).

**Table 2:** Comparison of high and low-UA groups, before and after PSM.

Variables	Before PSM			After PSM		
	UA (μmol/L)			UA (μmol/L)		
	≤ 320	> 320	*p*	≤ 320	> 320	*P*
N	1374	1119		686	686	
Follow years	24.61 ± 6.45	23.34 ± 7.52	< 0.001	23.77 ± 7.41	22.71 ± 7.94	0.011
Age (years)	42.25 ± 14.92	46.62 ± 14.82	< 0.001	45.64 ± 15.39	47.17 ± 15.84	0.070
Male, n (%)	1049 (76.35)	361 (32.26)	< 0.001	361 (52.60)	361 (52.60)	1.000
BMI (Kg/m^2^)	27.49 ± 6.59	30.7 ± 6.70	< 0.001	26.12 ± 5.39	31.52 ± 7.35	< 0.001
Waist-to-hip ratio	0.90 ± 0.09	0.97 ± 0.08	< 0.001	0.92 ± 0.09	0.96 ± 0.08	< 0.001
Death, n (%)	403 (29.33)	446 (39.86)	< 0.001	257 (37.50)	287 (41.80)	0.109
AST (U/L)	20.50 ± 9.28	24.71 ± 14.69	< 0.001	20.46 ± 7.16	24.75 ± 16.53	< 0.001
ALT (U/L)	17.27 ± 12.76	23.89 ± 17.62	< 0.001	16.69 ± 11.39	22.73 ± 16.99	< 0.001
eGFR (mL/min/1.73 m^2^)	86.53 ± 16.77	79.32 ± 13.85	< 0.001	83.97 ± 15.74	78.83 ± 14.29	< 0.001
Creatinine (μmol/L)	82.5 ± 13.74	95.88 ± 15.16	< 0.001	87.63 ± 14.46	92.00 ± 15.35	< 0.001
Urea nitrogen (mmol/L)	4.38 ± 1.40	5.07 ± 1.50	< 0.001	4.77 ± 1.49	4.94 ± 1.52	0.041
Race n (%)			< 0.001			0.317
Non-Hispanic White	392 (28.53)	409 (36.55)		233 (34.00)	244 (35.60)	
Non-Hispanic Black	348 (25.33)	307 (27.44)		180 (26.20)	201 (29.03)	
Mexican–American	567 (41.27)	359 (32.08)		244 (35.60)	213 (31.00)	
Other	67 (4.88)	44 (3.93)		29 (4.02)	28 (4.01)	
Total cholesterol (mmol/L)	5.12 ± 1.09	5.53 ± 1.14	< 0.001	5.20 ± 1.07	5.55 ± 1.19	< 0.001
Triglycerides (mmol/L)	1.53 ± 1.04	2.19 ± 1.55	< 0.001	1.48 ± 0.98	2.17 ± 1.54	< 0.001
HDL (mmol/L)	1.31 ± 0.37	1.14 ± 0.34	< 0.001	1.32 ± 0.38	1.17 ± 0.35	< 0.001
Hypertension, n (%)	554 (40.32)	716 (63.99)	< 0.001	300 (43.70)	451 (65.70)	< 0.001
Diabetes, n (%)	82 (20.35)	68 (15.28)	0.066	47 (18.30)	49 (17.10)	0.811
Fasting plasma glucose (mmol/L)	5.98 ± 3.05	5.71 ± 1.94	0.008	6.09 ± 3.19	5.76 ± 1.99	0.025
Glycated haemoglobin	5.81 ± 1.65	5.66 ± 1.01	0.006	5.86 ± 1.70	5.68 ± 0.98	0.019
UA (μmol/L)	249.23 ± 44.66	388.93 ± 55.69	< 0.001	254.37 ± 43.93	384.67 ± 54.88	< 0.001

*NAFLD* non-alcoholic fatty liver disease, *UA* uric acid, *NHANES III* Third National Health and Nutrition Examination Survey, *eGFR* estimated glomerular filtration rate, *BMI* body mass index, *WHR* Waist-to-hip ratio, *FPG* fasting plasma glucose, *HBA1C* glycated haemoglobin, *TCHO* total cholesterol, *TG* triglycerides, *HDL* high-density lipoprotein cholesterol, *CR* creatinine, *BUN* blood urea nitrogen, *AST* aspartate transaminase, *ALT* alanine aminotransferase.

### Time-dependent cox regression analysis

As the risk of mortality may change over time, and participants with high UA levels were significantly older at baseline than those with low UA levels, we used time-dependent Cox regression to minimise the bias of time-dependent exposure. The results in Supplementary Table 1 showed that age (HR 1.016, 95%CI 1.004–1.027, *P* = 0.006) and glycated haemoglobin (HR 1.078, 95%CI 1.029–1.130, *P* = 0.002) were significant predictive factors for mortality, while UA (as a continuous variable, HR 1.000, 95%CI 1.000–1.000, *P* = 0.495 and as a dichotomous variable, HR 0.997, 95%CI 0.998–1.006, *P* = 0.508) was still not an independent predictor. Sex subgroup analysis showed that UA level was not a significant risk factor in either the male (HR 1.000, 95%CI 1.000–1.000, *P* = 0.659) or female subgroups (HR 1.000, 95%CI 1.000–1.000, *P* = 0.647) (Supplementary Table 2). In multivariate regression, UA level was not independently associated with outcomes in all three groups (Supplementary Table 3), which was consistent with the outcome without classifying patients according to the severity of fatty liver.

### Propensity score matching

To further balance the baseline characteristics, we used PSM to match the age and sex between the two UA groups. The characteristics of the high and low-UA groups after PSM are presented in Table 2. The Kaplan–Meier curve is presented in Fig. 2 B. After matching for age and sex, survival probability increased in the low versus high UA group on the Kaplan–Meier curve (*P* < 0.05). On the time-dependent Cox regression, there was no independent association between higher UA (as a continuous variable HR 1.000, 95%CI 1.000–1.000, *P* = 0.554 and as a dichotomous variable, HR 1.005, 95%CI 0.994–1.015, *P* = 0.413) and mortality (Table 3).

**Table 3:** Time-dependent Cox regression with UA as a continuous or categorical variable for NAFLD mortality, after PSM.

Variables	Time-dependent cox regression			Time-dependent cox regression		
	HR	HR (95%CI)	*P*	HR	HR (95%CI)	*P*
UA level	1.000	1.000–1.000	0.554			
UA > 320 μmol/L				1.005	0.994–1.015	0.413
Age	1.024	1.009–1.038	0.001	1.024	1.009–1.038	0.001
Gender	1.199	0.790–1.819	0.395	1.216	0.799–1.852	0.361
Race	0.953	0.860–1.057	0.362	0.954	0.860–1.058	0.373
BMI	1.003	0.988–1.017	0.731	1.002	0.988–1.017	0.762
Waist-to-hip ratio	0.481	0.110–2.100	0.330	0.469	0.107–2.057	0.316
Hypertension	0.913	0.747–1.117	0.378	0.913	0.746–1.117	0.375
Diabetes	1.010	0.793–1.287	0.933	1.008	0.792–1.285	0.946
Glycated haemoglobin	1.056	0.994–1.121	0.077	1.056	0.995–1.120	0.072
AST	1.006	0.997–1.015	0.174	1.006	0.997–1.014	0.190
Total cholesterol	0.959	0.881–1.045	0.340	0.958	0.880–1.044	0.328
Triglycerides	1.051	0.967–1.141	0.240	1.050	0.968–1.140	0.241
HDL	1.039	0.777–1.390	0.796	1.044	0.780–1.396	0.773
eGFR	1.004	0.987–1.022	0.631	1.004	0.987–1.022	0.634
Creatinine	1.008	0.991–1.025	0.353	1.008	0.991–1.025	0.368

*NAFLD* non-alcoholic fatty liver disease, *UA* uric acid, *NHANES III* Third National Health and Nutrition Examination Survey, *eGFR* estimated glomerular filtration rate, *BMI* body mass index, *WHR* Waist-to-hip ratio, *FPG* fasting plasma glucose, *HBA1C* glycated haemoglobin, *TCHO* total cholesterol, *TG* triglycerides, *HDL* high-density lipoprotein cholesterol, *CR* creatinine, *BUN* blood urea nitrogen, *AST* aspartate transaminase, *ALT* alanine aminotransferase.

### Cause-specific death

The Kaplan–Meier analysis showed there was different in high- versus low-UA groups (*P* < 0.001) for cardiovascular disease (CVD)-related death, whereas identified no difference between the high and low-UA groups for cancer-related deaths in terms of survival probability (*P* > 0.05) (Figs. 2C and 2D). Time-dependent Cox regression showed that UA was not an independent risk factor for mortality from CVD or cancer (all *P* values > 0.05, Supplementary Tables 4 and 5).

The overall summary of the association between UA levels and mortality is shown in Table 4.

**Table 4:** Overall result of time-dependent Cox regression with UA as a continuous or dichotomous variable.

Characteristics	HR	HR (95%CI)	*P*
All-cause mortality			
Before PSM			
Continuous variable UA	1.000	1.000 (1.000, 1.000)	0.495
UA > 320 μmol/L	0.997	0.998 (0.998–1.006)	0.508
Sex subgroup before PSM			
Male–continuous UA	1.000	1.000 (1.000, 1.000)	0.659
Female–continuous UA	1.000	1.000 (1.000, 1.000)	0.647
After PSM			
Continuous variable UA	1.000	1.000 (1.000, 1.000)	0.554
UA > 320 μmol/L	1.005	1.005 (0.994–1.015)	0.413
Cause-specific death			
Cardiovascular related death			
Continuous variable UA	1.000	1.000 (1.000–1.000)	0.666
UA > 320 μmol/L	0.999	0.999 (0.982–1.017)	0.923
Cancer related death			
Continuous variable UA	1.000	1.000 (1.000–1.000)	0.637
UA > 320 μmol/L	0.988	0.988 (0.970–1.006)	0.179

*NAFLD* non-alcoholic fatty liver disease, *UA* uric acid, *NHANES III* Third National Health and Nutrition Examination Survey, *eGFR* estimated glomerular filtration rate, *BMI* body mass index, *WHR* Waist-to-hip ratio, *FPG* fasting plasma glucose, *HBA1C* glycated haemoglobin, *TCHO* total cholesterol, *TG* triglycerides, *HDL* high-density lipoprotein cholesterol, *CR* creatinine, *BUN* blood urea nitrogen, *AST* aspartate transaminase, *ALT* alanine aminotransferase.

### Sensitivity analysis

We included overall population (cases with and without decreased eGFR) in this sensitivity analysis. The results of time-dependent COX regression, as presented in Supplementary Table 6, showed that UA level was not significantly related to all-cause mortality of NAFLD participants irrespective of eGFR levels (P > 0.05).

To exclude the impact of uric acid lowering drugs, we investigated the correlation between uric acid and the prognosis of NAFLD in individuals with or without uric acid treatment. The Kaplan–Meier curve is presented in Supplementary Fig. 1D,E. After excluding patients with uric acid treatment, survival probability increased in the low UA group (P < 0.05). This difference was not found in patients using uric acid treatment (P = 0.2). But the result of time-dependent cox regression, which had adjusted confounding factors, showed that UA was not independently risk factor for the prognosis of NAFLD in NAFLD without using uric acid treatment (P > 0.05) (Supplementary Table 7). And using uric acid treatment was not an independent risk factor for all-cause death in all population (Supplementary Table 8).

## Discussion

The main finding of this study was that although UA level was associated with more severe metabolic dysfunction in patients with NAFLD, it was not an independent risk factor for mortality without severe renal dysfunction.

Although NAFLD patients with high UA levels had a relatively higher mortality rate, UA level with NAFLD was not an independent factor for survival. Metabolic disorders may play a mediating role in the relationship between UA level and NAFLD outcome. Numerous studies have indicated that increased UA levels are associated with metabolic syndrome^12^, CVD^13^, hypertension^14^, type 2 diabetes^15^, and hypertriglyceridemia^16^. The underlying mechanism among high UA levels, NAFLD, and metabolic syndrome may be insulin resistance caused by hyperuricaemia. Increased UA levels lead to endothelial dysfunction, oxidative stress, insulin resistance, and inflammation^17^. Insulin resistance is a key factor in the development of NAFLD^18^ and other metabolic diseases^19–21^. In addition, insulin resistance can promote lipolysis in adipocytes, which increases hepatic triglyceride accumulation and leads to hepatic steatosis^18^. In this study, the high-UA group had significantly more severe metabolic dysfunction than the low-UA group, which is consistent with previous studies^22^. After adjustment for the above metabolic profiles, the effect of UA level was no longer significant. The complex interaction between UA and metabolic syndrome may contribute to the association between UA levels and NAFLD prognosis.

At least 70% of the daily UA production is excreted by the kidney^23^. To reduce the effect of renal dysfunction, we excluded persons whose eGFR was below 60 mL/min/1.73 m^2^. By excluding patients whose eGFR was < 60 mL/min/1.73 m^2^, the influence of renal dysfunction on the results was minimised. But the association between UA and long-term outcomes of NAFLD was not statistically significant, and this result was not dependent on the baseline renal function level, as the sensitivity analysis have included patients with decreased eGFR. To reduce and evaluate the effect of uric acid-lowering drugs to renal function, which may result in an underestimation of mortality, we have performed multivariate regression with different GFR levels and the results kept unchanged.

Sex differences in the relationship between UA levels and death are controversial. A previous study reported that serum UA level was a predictor of all-cause, CVD, and cancer mortality in men^24^. However, another study reported that both low and high UA levels were predictive of increased mortality in both men and women^25^. In this study, UA was not identified as an independent risk factor for all-cause, CVD, or cancer mortality. A previous study showed that among patients with eGFR < 60 mL/min/1.73 m^2^, men had worse prognosis than women^26^. To reduce the effect of renal dysfunction, we have excluded persons whose eGFR was below 60 mL/min/1.73 m^2^. The different inclusion criteria of this study may explain the different results regarding sex disparities. Previous surveys showed that severity of NAFLD were associated with mortality^27^. In this study, UA level was not independently associated with outcomes in all three groups based on the grade of fatty liver, which was in line with Angulo et al. study^28^.

This study included a larger general population and a longer follow-up period; therefore, the results were relatively robust compared with those of other studies. This study had some limitations. First, the use of UA-lowering drugs was not available in the original database; thus, we were unable to analyse the effects of medication on UA levels. Second, UA levels were tested only at the beginning of the survey. The changes in UA levels and their effects on survival remain unclear.

In conclusion, UA level was not associated with long-term survival in patients with NAFLD without decreased eGFR.

### Supplementary Information

Supplementary Figure 1.

Supplementary Tables.

## Data Availability

The datasets are available online at https://www.cdc.gov/nchs/nhanes/index.htm.

## Abbreviations

NAFLD: Non-alcoholic fatty liver disease
UA: Uric acid
NHANES III: Third National Health and Nutrition Examination Survey
eGFR: Estimated glomerular filtration rate
BMI: Body mass index
WHR: Waist-to-hip ratio
FPG: Fasting plasma glucose
HBA1C: Glycated haemoglobin
TCHO: Total cholesterol
TG: Triglycerides
HDL: High-density lipoprotein cholesterol
CR: Creatinine
BUN: Blood urea nitrogen
AST: Aspartate transaminase
ALT: Alanine aminotransferase
