# Supplementary material for: Serum uric acid levels and prognosis of patients with non-alcoholic fatty liver disease
Source: Sci Rep. 2024 Mar 11;14:5923. doi: 10.1038/s41598-024-55845-5 (PMC10928212; doi:10.1038/s41598-024-55845-5)

**Supplementary Figure**

Supplementary Figure 1A Kaplan-Meier curve for overall death in mild fatty liver population.

Supplementary Figure 1B Kaplan-Meier curve for overall death in moderate fatty liver population.

Supplementary Figure 1C Kaplan-Meier curve for overall death in severe fatty liver population.

Supplementary Figure 1D: Kaplan-Meier curve for overall death after excluding uric acid treatment in overall population.

Supplementary Figure 1E: Kaplan-Meier curve for overall death in patients with uric acid treatment.


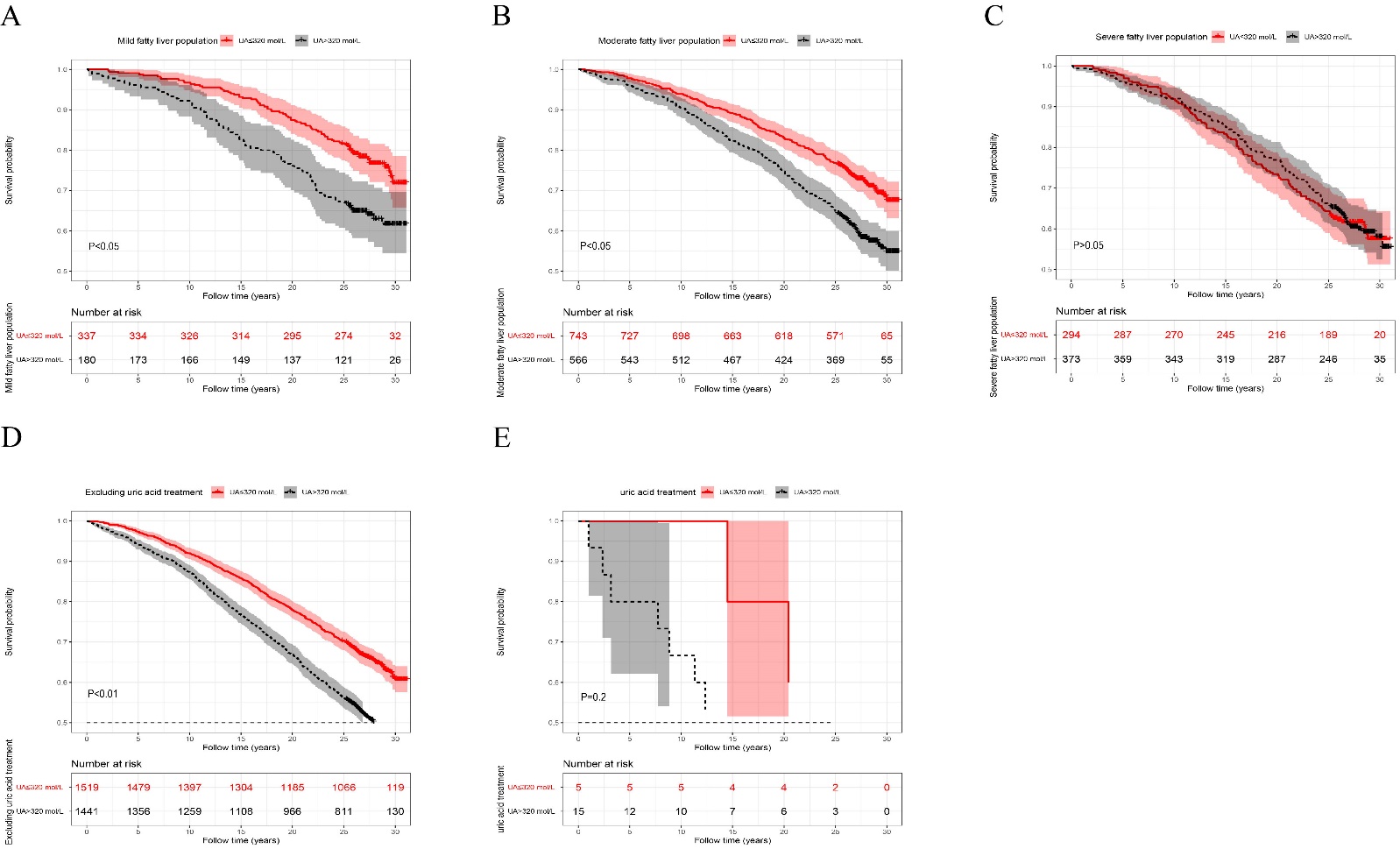

Supplement: Supplementary file 1 — Supplementary Figure 1. [file 41598_2024_55845_MOESM1_ESM.docx]
