# Supplementary material for: Serum uric acid levels and prognosis of patients with non-alcoholic fatty liver disease
Source: Sci Rep. 2024 Mar 11;14:5923. doi: 10.1038/s41598-024-55845-5 (PMC10928212; doi:10.1038/s41598-024-55845-5)
Supplement: Supplementary file 2 — Supplementary Tables. [file 41598_2024_55845_MOESM2_ESM.docx]

## The association between serum acid levels and outcome of patients with non-alcoholic fatty liver disease

**Supplementary Tables**

Supplementary Table 1: Time-dependent Cox regression with UA as a continuous or categorical variable for NAFLD mortality, before PSM.

Supplementary Table 2: Time-dependent Cox regression according to sex for the mortality of NALFD.

Supplementary table 3: Time-dependent Cox regression with UA as a continuous for NAFLD mortality in mild, moderate, and severe fatty group respectively.

Supplementary Table 4: Time-dependent Cox regression with UA as a continuous or dichotomous variable for CVD mortality.

Supplementary Table 5: Time-dependent Cox regression with UA as a continuous or dichotomous variable for cancer mortality.

Supplementary Table 6: Time-dependent Cox regression with UA as a continuous or dichotomous variable for all-cause mortality.

Supplementary table 7: Time-dependent Cox regression with UA for NAFLD mortality without uric acid treatment patients.

Supplementary table 8: Time-dependent Cox regression with UA for NAFLD mortality regardless of GFR levels.

Supplementary Table 1: Time-dependent Cox regression with UA as a continuous or categorical variable for NAFLD mortality, before PSM.

|  | **Time-dependent cox regression** | | |  | **Time-dependent cox regression** | | |
| --- | --- | --- | --- | --- | --- | --- | --- |
| **Variables** | **HR** | **HR (95%CI)** | ***P*** |  | **HR** | **HR (95%CI)** | ***P*** |
| UA | 1.000 | 1.000-1.000 | 0.495 |  |  |  |  |
| UA>320μmol/L |  |  |  |  | 0.997 | 0.998-1.006 | 0.508 |
| Age | 1.016 | 1.004-1.027 | 0.006 |  | 1.016 | 1.004-1.027 | 0.006 |
| Gender | 1.113 | 0.796-1.558 | 0.530 |  | 1.113 | 0.795-1.556 | 0.533 |
| Race | 0.923 | 0.852-1.001 | 0.052 |  | 0.923 | 0.851-1.000 | 0.050 |
| BMI | 0.997 | 0.985-1.009 | 0.617 |  | 0.997 | 0.985-1.008 | 0.587 |
| Waist-to-hip ratio | 0.710 | 0.218-2.313 | 0.569 |  | 0.709 | 0.218-2.313 | 0.569 |
| Hypertension | 0.959 | 0.821-1.120 | 0.594 |  | 0.959 | 0.821-1.121 | 0.602 |
| Diabetes | 0.962 | 0.793-1.168 | 0.696 |  | 0.959 | 0.790-1.164 | 0.670 |
| Glycated haemoglobin | 1.078 | 1.029-1.130 | 0.002 |  | 1.079 | 1.031-1.130 | 0.001 |
| AST | 1.003 | 0.996-1.009 | 0.405 |  | 1.003 | 0.996-1.009 | 0.403 |
| Total Cholesterol | 0.949 | 0.886-1.016 | 0.132 |  | 0.949 | 0.887-1.017 | 0.139 |
| Triglycerides | 1.066 | 1.005-1.131 | 0.035 |  | 1.065 | 1.004-1.129 | 0.038 |
| HDL | 1.129 | 0.900-1.415 | 0.294 |  | 1.127 | 0.899-1.413 | 0.300 |
| eGFR | 1.000 | 0.987-1.013 | 0.967 |  | 1.000 | 0.987-1.013 | 0.972 |
| creatinine | 1.003 | 0.990-1.017 | 0.610 |  | 1.003 | 0.990-1.017 | 0.618 |

NAFLD: non-alcoholic fatty liver disease; UA: uric acid; NHANES III: Third National Health and Nutrition Examination Survey; eGFR: estimated glomerular filtration rate; BMI: body mass index; WHR: Waist-to-hip ratio; FPG: fasting plasma glucose; HBA1C: glycated haemoglobin; TCHO: total cholesterol; TG: triglycerides; HDL: high-density lipoprotein cholesterol; CR: creatinine; BUN: blood urea nitrogen; AST: aspartate transaminase; ALT: alanine aminotransferase.

Supplementary Table 2: Time-dependent Cox regression according to sex for the mortality of NALFD.

| **Gender** | **Variables** | **HR** | **HR (95%CI)** | ***P*** |
| --- | --- | --- | --- | --- |
| **Male** | UA | 1.000 | 1.000-1.000 | 0.659 |
|  | Age | 1.019 | 1.000-1.037 | 0.047 |
|  | Race | 0.949 | 0.838-1.074 | 0.406 |
|  | BMI | 0.993 | 0.979-1.008 | 0.366 |
|  | Waist-to-hip ratio | 0.793 | 0.161-3.917 | 0.776 |
|  | Hypertension | 0.934 | 0.731-1.192 | 0.582 |
|  | Diabetes | 0.998 | 0.764-1.303 | 0.988 |
|  | glycated haemoglobin | 1.072 | 0.998-1.151 | 0.055 |
|  | AST | 1.000 | 0.990-1.009 | 0.929 |
|  | Total Cholesterol | 0.941 | 0.855-1.036 | 0.215 |
|  | Triglycerides | 1.109 | 1.023-1.202 | 0.012 |
|  | HDL | 1.192 | 0.864-1.643 | 0.284 |
|  | eGFR | 1.011 | 0.991-1.031 | 0.286 |
|  | creatinine | 1.021 | 0.997-1.046 | 0.085 |
| **Female** | UA | 1.000 | 1.000-1.000 | 0.647 |
|  | Age | 1.013 | 0.998-1.029 | 0.096 |
|  | Race | 0.916 | 0.820-1.023 | 0.119 |
|  | BMI | 0.999 | 0.978-1.021 | 0.949 |
|  | Waist-to-hip ratio | 0.536 | 0.076-3.787 | 0.532 |
|  | Hypertension | 0.979 | 0.791-1.213 | 0.847 |
|  | Diabetes | 0.951 | 0.713-1.269 | 0.734 |
|  | glycated haemoglobin | 1.094 | 1.022-1.171 | 0.009 |
|  | AST | 1.007 | 0.997-1.016 | 0.157 |
|  | Total Cholesterol | 0.970 | 0.873-1.077 | 0.569 |
|  | Triglycerides | 1.020 | 0.935-1.113 | 0.654 |
|  | HDL | 1.106 | 0.794-1.541 | 0.552 |
|  | eGFR | 0.988 | 0.968-1.008 | 0.240 |
|  | creatinine | 0.991 | 0.973-1.010 | 0.362 |

NAFLD: non-alcoholic fatty liver disease; UA: uric acid; NHANES III: Third National Health and Nutrition Examination Survey; eGFR: estimated glomerular filtration rate; BMI: body mass index; WHR: Waist-to-hip ratio; FPG: fasting plasma glucose; HBA1C: glycated haemoglobin; TCHO: total cholesterol; TG: triglycerides; HDL: high-density lipoprotein cholesterol; CR: creatinine; BUN: blood urea nitrogen; AST: aspartate transaminase; ALT: alanine aminotransferase.

Supplementary table 3: Time-dependent Cox regression with UA as a continuous for NAFLD mortality in mild, moderate, and severe fatty group respectively.

|  | Time-dependent cox regression | | | | | | | | |
| --- | --- | --- | --- | --- | --- | --- | --- | --- | --- |
|  | Mild fatty liver | | | Moderate fatty liver | | | Severe fatty liver | | |
| Variables | HR | HR (95%CI) | P | HR | HR (95%CI) | P | HR | HR (95%CI) | P |
| UA | 1.000 | 1.000-1.000 | 0.580 | 1.000 | 1.000-1.000 | 0.544 | 1.000 | 1.000-1.000 | 0.054 |
| Age | 1.117 | 1.084-1.112 | 0.000 | 1.012 | 0.996-1.028 | 0.132 | 1.016 | 0.995-1.037 | 0.132 |
| Gender | 0.789 | 0.325-1.941 | 0.600 | 1.186 | 0.742-1.896 | 0.477 | 1.077 | 0.569-2.041 | 0.819 |
| Race | 0.914 | 0.715-1.168 | 0.470 | 0.974 | 0.871-1.088 | 0.637 | 0.945 | 0.818-1.092 | 0.440 |
| BMI | 1.023 | 0.981-1.068 | 0.290 | 0.997 | 0.980-1.014 | 0.696 | 0.993 | 0.971-1.016 | 0.566 |
| Hypertension | 1.074 | 0.675-1.707 | 0.760 | 0.943 | 0.756-1.177 | 0.606 | 1.075 | 0.803-1.438 | 0.629 |
| Diabetes | 0.949 | 0510-1.765 | 0.870 | 0.953 | 0.722-1.257 | 0.731 | 0.854 | 0.618-1.181 | 0.341 |
| Glycated haemoglobin | 1.183 | 0.993-1.410 | 0.060 | 1.062 | 0.989-1.141 | 0.096 | 1.082 | 1.006-1.165 | 0.035 |
| AST | 1.011 | 0.990-1.032 | 0.300 | 1.007 | 0.996-1.018 | 0.204 |  |  |  |
| ALT |  |  |  |  |  |  | 0.995 | 0.986-1.004 | 0.319 |
| Total Cholesterol | 1.218 | 0.999-1.410 | 0.050 |  |  |  | 1.055 | 0.932-1.195 | 0.395 |
| Triglycerides | |  |  | 1.057 | 0.981-1.139 | 0.142 | 0.983 | 0.877-1.103 | 0.776 |
| HDL | 0.483 | 0.264-0.882 | 0.020 | 1.064 | 0.787-1.439 | 0.686 |  |  |  |
| eGFR | 1.058 | 1.021-1.097 | 0.000 | 0.992 | 0.974-1.011 | 0.427 | 1.009 | 0.982-1.037 | 0.525 |
| BUN |  |  |  |  |  |  | 1.033 | 0.942-1.131 | 0.493 |
| CR | 1.039 | 1.002-1.078 | 0.040 | 0.999 | 0.980-1.011 | 0.877 | 1.009 | 0.981-1.038 | 0.532 |
| WHR | 0.770 | 0.029-20.225 | 0.880 | 0.673 | 0.128-3.545 | 0.640 |  |  |  |

NAFLD: non-alcoholic fatty liver disease; UA: uric acid; NHANES III: Third National Health and Nutrition Examination Survey; eGFR: estimated glomerular filtration rate; BMI: body mass index; WHR: Waist-to-hip ratio; FPG: fasting plasma glucose; HBA1C: glycated haemoglobin; TCHO: total cholesterol; TG: triglycerides; HDL: high-density lipoprotein cholesterol; CR: creatinine; BUN: blood urea nitrogen; AST: aspartate transaminase; ALT: alanine aminotransferase.

Supplementary Table 4: Time-dependent Cox regression with UA as a continuous or dichotomous variable for CVD mortality.

|  | **Time-dependent cox regression** | | |  | **Time-dependent cox regression** | | |
| --- | --- | --- | --- | --- | --- | --- | --- |
| **Variables** | **HR** | **HR (95%CI)** | ***P*** |  | **HR** | **HR (95%CI)** | ***P*** |
| UA | 1.000 | 1.000-1.000 | 0.666 |  |  |  |  |
| UA>320μmol/L |  |  |  |  | 0.999 | 0.982-1.017 | 0.923 |
| Age | 1.006 | 0.984-1.029 | 0.586 |  | 1.006 | 0.983-1.029 | 0.612 |
| Gender | 1.373 | 0.701-2.690 | 0.356 |  | 1.365 | 0.697-2.673 | 0.364 |
| Race | 0.966 | 0.823-1.133 | 0.667 |  | 0.966 | 0.824-1.133 | 0.671 |
| BMI | 1.005 | 0.982-1.028 | 0.667 |  | 1.004 | 0.981-1.027 | 0.733 |
| Waist-to-hip ratio | 1.531 | 0.140-16.725 | 0.727 |  | 1.525 | 0.139-16.674 | 0.730 |
| Hypertension | 1.325 | 0.951-1.847 | 0.096 |  | 1.324 | 0.950-1.846 | 0.098 |
| Diabetes | 0.723 | 0.491-1.065 | 0.101 |  | 0.724 | 0.491-1.067 | 0.102 |
| Glycated haemoglobin | 1.174 | 1.083-1.272 | 0.000 |  | 1.179 | 1.089-1.275 | 0.000 |
| AST | 0.996 | 0.982-1.010 | 0.576 |  | 0.996 | 0.982-1.010 | 0.568 |
| Total Cholesterol | 1.009 | 0.884-1.152 | 0.892 |  | 1.007 | 0.881-1.151 | 0.921 |
| Triglycerides | 0.962 | 0.850-1.090 | 0.545 |  | 0.960 | 0.848-1.086 | 0.515 |
| HDL | 0.675 | 0.407-1.119 | 0.128 |  | 0.677 | 0.408-1.123 | 0.131 |
| eGFR | 0.987 | 0.961-1.014 | 0.352 |  | 0.987 | 0.961-1.014 | 0.340 |
| creatinine | 0.993 | 0.967-1.020 | 0.627 |  | 0.993 | 0.966-1.020 | 0.588 |

NAFLD: non-alcoholic fatty liver disease; UA: uric acid; NHANES III: Third National Health and Nutrition Examination Survey; eGFR: estimated glomerular filtration rate; BMI: body mass index; WHR: Waist-to-hip ratio; FPG: fasting plasma glucose; HBA1C: glycated haemoglobin; TCHO: total cholesterol; TG: triglycerides; HDL: high-density lipoprotein cholesterol; CR: creatinine; BUN: blood urea nitrogen; AST: aspartate transaminase; ALT: alanine aminotransferase.

Supplementary Table 5: Time-dependent Cox regression with UA as a continuous or dichotomous variable for cancer mortality.

| **Variables** | **HR** | **HR (95%CI)** | ***P*** |  | **HR** | **HR (95%CI)** | ***P*** |
| --- | --- | --- | --- | --- | --- | --- | --- |
| UA | 1.000 | 1.000-1.000 | 0.637 |  |  |  |  |
| UA>320 μmol/L |  |  |  |  | 0.988 | 0.970-1.006 | 0.179 |
| Age | 1.066 | 1.053-1.079 | 0.000 |  | 1.065 | 1.053-1.079 | 0.000 |
| Gender | 0.920 | 0.586-1.445 | 0.718 |  | 0.940 | 0.600-1.472 | 0.786 |
| Race | 0.835 | 0.717-0.972 | 0.020 |  | 0.833 | 0.715-0.970 | 0.019 |
| Hypertension | 0.844 | 0.624-1.142 | 0.272 |  | 0.850 | 0.629-1.149 | 0.292 |
| Glycated haemoglobin | 1.021 | 0.920-1.133 | 0.693 |  | 1.015 | 0.916-1.125 | 0.773 |
| AST | 0.998 | 0.983-1.013 | 0.767 |  | 0.998 | 0.984-1.013 | 0.812 |
| BMI | 0.990 | 0.964-1.017 | 0.458 |  | 0.992 | 0.966-1.018 | 0.526 |
| Waist-to-hip ratio | 7.720 | 0.810-73.539 | 0.076 |  | 8.480 | 0.883-81.395 | 0.064 |
| Total Cholesterol | 0.976 | 0.847-1.125 | 0.742 |  | 0.980 | 0.850-1.130 | 0.782 |
| Triglycerides | 0.984 | 0.868-1.115 | 0.798 |  | 0.988 | 0.872-1.119 | 0.845 |
| HDL | 0.765 | 0.475-1.230 | 0.269 |  | 0.763 | 0.474-1.227 | 0.264 |
| creatinine | 1.006 | 0.993-1.019 | 0.387 |  | 1.007 | 0.993-1.020 | 0.319 |

NAFLD: non-alcoholic fatty liver disease; UA: uric acid; NHANES III: Third National Health and Nutrition Examination Survey; eGFR: estimated glomerular filtration rate; BMI: body mass index; WHR: Waist-to-hip ratio; FPG: fasting plasma glucose; HBA1C: glycated haemoglobin; TCHO: total cholesterol; TG: triglycerides; HDL: high-density lipoprotein cholesterol; CR: creatinine; BUN: blood urea nitrogen; AST: aspartate transaminase; ALT: alanine aminotransferase.

Supplementary Table 6: Time-dependent Cox regression with UA as a continuous or dichotomous variable for NAFLD mortality in all patients.

|  | **Time-dependent cox regression** | | |  | **Time-dependent cox regression** | | |
| --- | --- | --- | --- | --- | --- | --- | --- |
| **Variables** | **HR** | **HR (95%CI)** | **P** |  | **HR** | **HR (95%CI)** | P |
| UA level | 1.000 | 1.000-1.000 | 0.673 |  |  |  |  |
| UA>320μmol/L |  |  |  |  | 0.997 | 0.990-1.005 | 0.486 |
| Gender | 1.166 | 1.010-1.346 | 0.036 |  | 1.191 | 1.036-1.370 | 0.014 |
| Age | 1.077 | 1.070-1.084 | 0.000 |  | 1.077 | 1.070-1.083 | 0.000 |
| Race | 0.879 | 0.826-0.936 | 0.000 |  | 0.878 | 0.825-0.935 | 0.000 |
| BMI | 1.004 | 0.993-1.014 | 0.519 |  | 1.005 | 0.994-1.015 | 0.391 |
| triglycerides | 1.024 | 0.979-1.071 | 0.298 |  | 1.027 | 0.982-1.037 | 0.244 |
| Waist-to-hip ratio | 7.078 | 2.948-16.994 | 0.000 |  | 7.3 | 3.032-17.578 | 0.000 |
| HDL | 1.108 | 0.928-1.325 | 0.257 |  | 1.105 | 0.925-1.321 | 0.270 |
| Hypertension | 1.113 | 0.978-1.267 | 0.104 |  | 1.119 | 0.983-1.273 | 0.089 |
| eGFR | 0.996 | 0.991-1.000 | 0.074 |  | 0.995 | 0.990-1.000 | 0.038 |
| AST | 0.998 | 0.992-1.004 | 0.494 |  | 0.998 | 0.992-1.004 | 0.545 |
| Glycated haemoglobin | 1.158 | 1.121-1.196 | 0.000 |  | 1.154 | 1.117-1.191 | 0.000 |

NAFLD: non-alcoholic fatty liver disease; UA: uric acid; NHANES III: Third National Health and Nutrition Examination Survey; eGFR: estimated glomerular filtration rate; BMI: body mass index; WHR: Waist-to-hip ratio; FPG: fasting plasma glucose; HBA1C: glycated haemoglobin; TCHO: total cholesterol; TG: triglycerides; HDL: high-density lipoprotein cholesterol; CR: creatinine; BUN: blood urea nitrogen; AST: aspartate transaminase; ALT: alanine aminotransferase.

Supplementary table 7: Time-dependent Cox regression with UA for NAFLD mortality without uric acid treatment patients.

|  | Time-dependent cox regression | | |
| --- | --- | --- | --- |
| Variables | HR | HR (95%CI) | P |
| UA | 1 | 1.000-1.000 | 0.553 |
| Age | 1.097 | 1.086-1.108 | <0.001 |
| Gender | 0.623 | 0.450-0.862 | 0.004 |
| Race | 0.863 | 0.799-0.932 | <0.001 |
| BMI | 1 | 0.987-1.012 | 0.943 |
| Hypertension | 0.984 | 0.842-1.150 | 0.837 |
| Diabetes | 1.185 | 0.991-1.417 | 0.062 |
| Glycated haemoglobin | 1.105 | 1.054-1.160 | <0.001 |
| AST | 0.999 | 0.993-1.006 | 0.803 |
| Total Cholesterol | 1.001 | 0.935-1.071 | 0.978 |
| WHR | 13.991 | 4.713-41.538 | <0.001 |
| CR | 1.028 | 1.015-1.042 | <0.001 |
| Triglycerides | 1.012 | 0.956-1.072 | 0.674 |
| HDL | 1.126 | 0.899-1.410 | 0.302 |
| eGFR | 1.034 | 1.021-1.047 | <0.001 |

NAFLD: non-alcoholic fatty liver disease; UA: uric acid; NHANES III: Third National Health and Nutrition Examination Survey; eGFR: estimated glomerular filtration rate; BMI: body mass index; WHR: Waist-to-hip ratio; FPG: fasting plasma glucose; HBA1C: glycated haemoglobin; TCHO: total cholesterol; TG: triglycerides; HDL: high-density lipoprotein cholesterol; CR: creatinine; BUN: blood urea nitrogen; AST: aspartate transaminase; ALT: alanine aminotransferase.

Supplementary table 8: Time-dependent Cox regression with UA for NAFLD mortality regardless of GFR levels.

|  | Time-dependent cox regression | | |
| --- | --- | --- | --- |
| Variables | HR | HR (95%CI) | *P* |
| UA | 1.000 | 1.000-1.000 | 0.640 |
| Age | 1.076 | 1.070-1.083 | <0.001 |
| Gender | 1.059 | 0.970-1.236 | 0.468 |
| Race | 0.886 | 0.832-0.943 | <0.001 |
| BMI | 1.004 | 0.993-1.015 | 0.475 |
| Hypertension | 1.008 | 0.955-1.239 | 0.203 |
| Diabetes | 1.247 | 1.082-1.473 | 0.002 |
| Glycated haemoglobin | 1.117 | 1.076-1.160 | <0.001 |
| AST | 0.998 | 0.993-1.004 | 0.580 |
| Total Cholesterol | 1.002 | 0.947-1.060 | 0.952 |
| WHR | 6.692 | 2.776-16.131 | <0.001 |
| CR | 1.006 | 1.004-1.009 | <0.001 |
| Uric acid treatment | 1.340 | 0.828-2.169 | 0.234 |
| Triglycerides | 1.015 | 0.967-1.066 | 0.541 |
| HDL | 1.105 | 0.919-1.330 | 0.289 |
| eGFR | 1.092 | 0.936-1.247 | 0.264 |

NAFLD: non-alcoholic fatty liver disease; UA: uric acid; NHANES III: Third National Health and Nutrition Examination Survey; eGFR: estimated glomerular filtration rate; BMI: body mass index; WHR: Waist-to-hip ratio; FPG: fasting plasma glucose; HBA1C: glycated haemoglobin; TCHO: total cholesterol; TG: triglycerides; HDL: high-density lipoprotein cholesterol; CR: creatinine; BUN: blood urea nitrogen; AST: aspartate transaminase; ALT: alanine aminotransferase.
